# Consumption of Bilberries Controls Gingival Inflammation 

**DOI:** 10.3390/ijms160510665

**Published:** 2015-05-11

**Authors:** Cecilia Widén, Michael Coleman, Sladjana Critén, Pernilla Karlgren-Andersson, Stefan Renvert, G. Rutger Persson

**Affiliations:** 1 School of Health & Society, University of Kristianstad, SE-29188 Kristianstad, Sweden; E-Mails: cecilia.widen@hkr.se (C.W.); sladjana.criten@hkr.se (S.C.); pernilla.karlgren_andersson@hkr.se (P.K.-A.); stefan.renvert@hkr.se (S.R.); 2 School of Life and Health Sciences, Aston University, B4 7ET Birmingham, UK; E-Mail: m.d.coleman@aston.ac.uk; 3 Blekinge Institute of Technology, SE-37179 Karlskrona, Sweden; 4 Department of Restorative Dentistry and Periodontology, Trinity College, Dublin Dental University Hospital, 2 Dublin, Ireland; 5 School of Dentistry, Department of Periodontics, University of Washington, Seattle, WA 98195, USA

**Keywords:** bleeding, clinical study, cytokines, gingival crevicular fluid, human

## Abstract

Bioactive molecules in berries may be helpful in reducing the risk of oral diseases. The aim of this study was to determine the effect of bilberry consumption on the outcome of a routine dental clinical parameter of inflammation, bleeding on probing (BOP), as well as the impact on selected biomarkers of inflammation, such as cytokines, in gingival crevicular fluid (GCF) in individuals with gingivitis. Study individuals who did not receive standard of care treatment were allocated to either a placebo group or to groups that consumed either 250 or 500 g bilberries daily over seven days. The placebo group consumed an inactive product (starch). A study group, receiving standard of care (debridement only) was also included to provide a reference to standard of care treatment outcome. Cytokine levels were assayed using the Luminex MagPix system. The mean reduction in BOP before and after consumption of test product over 1 week was 41% and 59% in the groups that consumed either 250 or 500 g of bilberries/day respectively, and was 31% in the placebo group, and 58% in the standard of care reference group. The analysis only showed a significant reduction in cytokine levels in the group that consumed 500 g of bilberries/day. A statistically significant reduction was observed for IL-1β (*p* = 0.025), IL-6 (*p* = 0.012) and VEGF (*p* = 0.017) in GCF samples in the group that consumed 500 g of bilberries daily. It appears that berry intake has an ameliorating effect on some markers of gingival inflammation reducing gingivitis to a similar extent compared to standard of care.

## 1. Introduction

Gingivitis (bleeding from the gums) is a common clinical feature and as many as 50% of teenagers in Sweden may have gingivitis [1]. This condition presents as swelling, redness and bleeding at the gums and is an inflammatory reaction driven by the release of pro-inflammatory cytokines, although it is reversible [2]. Assessment of the frequency of gingival bleeding is the standard method to diagnose gingivitis, and is referred to as “bleeding on probing” (BOP). As a result of the local gingival inflammatory response, an exudate (gingival crevicular fluid, GCF) from the area between the tooth and the gum tissues effectively reflects serum composition, including the presence of cytokines [3], which modulate the balance between humoral and cell-based immune responses. 

Good health is associated with adequate fruit and berry intake. There is a growing documentation on antioxidant [4,5,6], anti-inflammatory [7] and antibacterial [8] effects of berries, which is strongly associated with the phenolic content. Bilberries (*Vaccinium myrtillus*) contain compounds that may control inflammation [7]. A reduction in gingival inflammation and beneficial changes in the oral microbiota have been reported in humans on a restricted diet typical for the “Stone Age” where oral hygiene is maintained in the absence of tooth brushing [9]. Indeed, encapsulated fruit, vegetable and berry juice concentrates appear to improve gingival conditions [10]. 

The scope of the present study was to determine the outcome on oral inflammation, and levels of cytokines in GCF after the consumption of 250 or 500 g bilberries daily over seven days in individuals with gingivitis. The same biomarkers were also studied in a placebo group. 

## 2. Results and Discussion

Data from 24 adult individuals, 17 females (70.8%) and 7 males (29.2%), with gingivitis and a mean age of 28 years (S.D. ± 11) were included. Standard of care reference group: 8 adult individuals, 4 females (50%), 4 males (50%), age 50.5 years (S.D. ± 9.5). Baseline bleeding on probing 39.6% (S.D. ± 8.1) and after 6 months 17.1% (S.D ± 7.7). The mean reduction in bleeding on probing before and after consumption of test product was 41% in the group that consumed 250 g bilberries/day, 59% in individuals who consumed 500 g bilberries/day, and 31% in the placebo group (Table 1). The mean reduction in bleeding was 58% in the group that served as standard of care reference group receiving debridement and oral hygiene instructions only. Data analysis demonstrated that the change in BOP over the study period differed significantly between baseline and study endpoint in all groups. However, there was only a statistical change in BOP between the 500 g bilberry and placebo group and the reduction in BOP was unexpectedly high. While this observation might be explained in terms of “the Hawthorne effect”, where study individuals devote greater attention to a given task due to the attention being focused on them, in this report the individuals stated that they had not consciously changed their oral hygiene. The reduction of gingivitis (bleeding) from the berry intake with 500 g bilberry as reported was surprising but consistent with a report on the reduction in gingival inflammation, where individuals lived on a restricted diet without sugar, but rich in berry intake [9]. The present study also demonstrated that participating in a study such as in the present one resulted in improvement of gingival (gum) conditions that is greater than the Hawthorne effect. Thus, it is important to include not only a placebo control group but also a reference to the results of the “standard of care” treatment. The reduction in the extent of bleeding in the reference group reported here is consistent with many other reports and clinical experiences.

**Table 1:** Baseline and study end point clinical bleeding on probing (BOP) (%) in the bilberry, placebo and standard of care reference groups. (BL = baseline, EP = study endpoint, Ch = change, SOC = standard of care).

Individual	250 g Bilberry			500 g Bilberry			Placebo			SOC		
	BL (%)	EP (%)	Ch (%)	BL (%)	EP (%)	Ch (%)	BL (%)	EP (%)	Ch (%)	BL (%)	EP (%)	Ch (%)
1	13	6	54	34	18	47	37	15	59	53	18	66
2	23	11	52	48	33	31	54	54	0	32	14	56
3	28	17	39	30	8	73	25	25	0	43	27	37
4	25	14	44	29	15	48	39	26	33	33	4	88
5	33	21	36	24	8	67	31	21	32	33	12	64
6	21	11	48	39	16	59	26	14	46	36	20	44
7	23	19	17	39	10	74	31	16	48	37	15	59
8	23	15	35	25	7	72	26	18	31	50	27	46
mean	25	15	41	34	14	59	34	24	31	40	17	58

**Figure 1:**
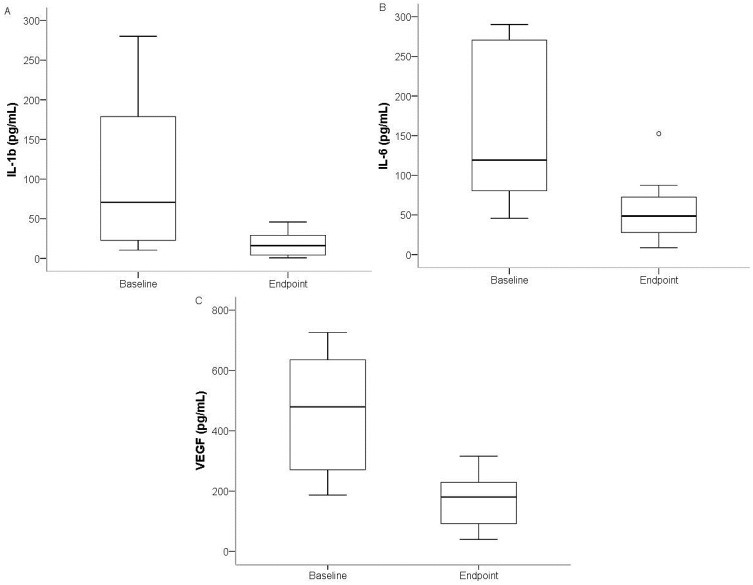
Boxplot diagram (median values, 25th and 75th percentiles, open circle represents outlier value) illustrating gingival crevicular fluid (GCF) changes in IL-1β (**A**); IL-6 (**B**); and VEGF (**C**) between baseline and at study endpoint in individuals who consumed 500 g bilberries daily.

No significant changes in cytokine levels were found in the placebo group or in the berry group that consumed 250 g of bilberries. The analysis identified significant differences between baseline and study endpoint after intake of 500 g of bilberries/day for three of the cytokines studied: interleukin (IL)-1β (*p* = 0.025), IL-6 (*p* = 0.012), and vascular endothelial growth factor (VEGF) (*p* = 0.017) (Figure 1A–C and Table 2).

Berry components may serve as bioactive molecules for the prevention and/or treatment of inflammatory diseases, including gingival inflammation. It remains to be established, as to the optimal degree of consumption and species of berries that may provide clinically measurable effects on inflammation [11]. The guidelines provided by the Swedish National Food Agency suggest a daily intake of 500 g fruits, berries and vegetables. We have identified that bilberries may be one of many berries that can be consumed on a daily basis at such amounts. Bleeding on probing was effectively reduced following intake of 500 g bilberries, whereas an intake of 250 g was not sufficient to reduce gingival inflammation. Standard of care resulted i similar changes in bleeding on probing as did the intake of 500 g bilberries. This average reduction (59%) among individuals consuming 500 g of bilberries is clinically relevant. In fact, this reduction exceeds the changes of gingival inflammation presented in many studies of experimental gingivitis and resolution of inflammation through the introduction of improved oral hygiene procedures [12,13,14]. The observed reduction is also remarkable considering the fact that none of the study individuals had severe inflammation at baseline. Based on our findings, daily intake of bilberries could potentially control gingival inflammation in the general population. It appears that there are no studies reporting adverse effects of long-term intake of bilberries at daily dosages used in the present study and none of the participants reported adverse effects related to the dietary intake of bilberries. Assuming that gingival inflammation is only one expression of inflammation, the positive impact on health could be important if bilberry consumption has similar effects on human inflammation in other tissues and organs. We also observed changes in serum cytokine levels but failed to identify that the intake of bilberries at 500 g level had any significant impact. It is possible that berry intake over seven days is not sufficient to induce systemic effects. Whilst the inflammatory changes in the present study reflect the impact of bilberry consumption in chronic inflammatory conditions, it is unknown as to whether cases of acute inflammation may respond in a similar fashion [7].

**Table 2:** Concentrations of IL-1β, IL-1ra, IL-6, IL-12, MIP-1α, PDGF, and VEGF in gingival crevicular fluid in the groups that consumed 250 or 500 g bilberries/day or placebo over seven days. Data are presented for median values, 25th and 75th percentile due to non-normal distribution. Exact *p*-values are presented.

Cytokine	Median pg/mL	25% pg/mL	75% pg/mL	Median pg/mL	25% pg/mL	75% pg/mL	Median Ratio	*p* Value
	250 g bilberry baseline			250 g bilberry study end point				
IL-1ra	0.7	0.2	6.9	0.4	0.0	35.8	1.8	0.715
IL-1β	18.0	10.7	144.2	29.6	6.2	146.0	0.6	0.715
IL-6	84.8	52.9	96.0	67.7	34.3	106.5	1.3	0.715
IL-12	0.8	0.8	1.4	0.8	0.6	1.4	1.0	0.317
IP-10	371.5	235.9	689.0	522.5	196.7	1392.2	0.7	0.735
MIP-1α	3.0	1.9	4.7	2.0	0.9	2.7	1.5	0.180
PDGF	0.0	0.0	0.0	0.2	0.0	1.7	0.0	0.068
VEGF	303.7	228.9	575.3	266.8	224.9	483.1	1.1	0.263
	500 g bilberry baseline			500 g bilberry study end point				
IL-1ra	0.5	0.1	2.9	0.2	0.0	0.6	2.5	0.310
IL-1β	70.6	21.8	220.5	16.1	4.1	31.9	4.4	0.025
IL-6	119.3	73.9	280.5	48.7	25.2	80.0	2.4	0.012
IL-12	1.5	1.2	2.3	0.7	0.6	1.9	2.1	0.310
IP-10	389.3	135.4	990.3	176.3	113.6	659.2	2.2	0.069
MIP-1α	3.2	1.8	7.1	1.1	0.8	2.7	2.9	0.107
PDGF	0.8	0.0	1.7	1.5	0.1	3.5	0.5	0.237
VEGF	479.6	250.6	680.5	180.6	87.0	238.6	2.7	0.017
	Placebo baseline			Placebo study end point				
IL-1β	70.9	21.8	196.5	30.5	23.4	88.1	2.3	0.674
IL-6	1.5	0.3	2.2	0.6	0.3	1.6	2.5	0.889
VEGF	330.9	299.6	592.4	305.1	188.7	723.2	1.1	0.889

The reduction in IL-1β may be reflective of the reported antibacterial action of bilberries [15]. Others have shown that supplementation with bilberry juice result in significant decrease in blood concentrations of IL-6 [16]. Furthermore, data have shown that daily bilberry consumption of 200 g bilberry puree and 40 g of dried bilberries over eight weeks tended to decrease blood IL-6 and IL-12 [7]. Elevated serum VEGF concentrations have been associated with gingival and periodontal inflammation [17]. VEGF may induce angiogenesis and plays a key role in periodontal disease progression and resolution [18]. The observed reduction in VEGF levels in GCF in the present study was in agreement with the clinically observed reduction in bleeding, which suggests that bilberries may have an effect on vascular inflammation. 

## 3. Experimental Section

### 3.1. Study Individuals 

In the present study, the guidelines by the Declaration of Helsinki were followed. The Regional Ethics Review Board in Lund, Sweden approved the study. All participating individuals signed written informed consent. The study was a randomized controlled trial based on healthy individuals who were ≥18 years of age. Individuals were excluded if they had been treated with systemic antibiotics in the preceding 3 months, and/or were prescribed anti-inflammatory medications on a daily basis. Pregnant women were also excluded. The study individuals were allocated to either a placebo group or to groups that consumed either 250 or 500 g bilberries daily over seven days. The placebo group consumed potato starch (Potatismjöl, Lyckeby, Sweden).

### 3.2. Bleeding on Probing 

Probing depth was assessed at six sites per tooth (PCR-12, Hu-Friedy, Chicago, IL, USA). Bleeding on probing (BOP) was assessed 30 s after probing. BOP was categorized as bleeding or no bleeding.

### 3.3. Sampling from Gingival Crevicular Fluid for Cytokine Analysis

Four sterile paper points (Periopaper, Oraflow Inc., New York, NY, USA) were inserted into selected pockets from the mesial surface of each first molar until resistance was met and left *in situ* during 30 s. The paper points were then placed into a labeled Eppendorf tube (1.5 mL natural flat cap microcentrifuge tubes, Starlab, Ahrensburg, Germany). The vials were stored in a freezer at −79 °C until processed for cytokine content.

### 3.4. Sampling from Blood for Cytokine Analysis 

Peripheral venous blood samples were drawn into SST II vials (BD Vacutainer, Oxford, UK). The vials were centrifuged for 10 min at 3000× *g* and 4 °C. Serum was harvested and transferred into a labeled Eppendorf tube (1.5 mL natural flat cap microcentrifuge tubes, Starlab, Ahrensburg, Germany). The vials were stored in a freezer at −79° C until processed for cytokine content.

### 3.5. Determination of Cytokines Using Bio-Plex Assays 

Cytokine analysis was performed according to Bio-Rad’s instructions for the xMAP technology with multiplex beads. Plates were measured using the Bio-Plex MagPix System and analyzed with the Bio-Plex Manager version 6.0 (Luminex, Austin, TX, USA). Antibody-coupled magnetic beads were added to each 96 well plate. The plates were washed with Bio-Plex wash buffer (2 × 100 µL). The content of the GCF samples were then pipetted and added to 96-well micro-plates with antibody-coupled beads. Samples were incubated (30 min) and washed with Bio-Plex wash buffer (3 × 100 µL) removing unbound protein. A 25 µL aliquot of 1 × concentration of Bio-Plex biotinylated detection antibody specific for a different epitope on the cytokine was added to each well, incubated (30 min), and subsequently washed with Bio-Plex wash buffer (3 × 100 µL). The reaction mixture was detected by streptavidin-phycoerythrin (streptavidin-PE). The assay was developed in a 50 µL aliquot of 1 × concentration of streptavidin-PE (10 min), followed by a Bio-Plex wash buffer (3 × 100 µL). Beads were re-suspended in each well with 125 µL of Bio-Plex assay buffer and shaken on a plate shaker (900 rpm, 30 s). The reaction was measured using fluorescently labeled reporter molecules associated with each target protein. Cytokine concentrations in the samples were calculated by Bio-Plex software using a standard curve derived from a recombinant cytokine standard, included in the 96-well plate. The following cytokines were studied: IL-1β, IL-1ra, IL-6, IL-12, interferon gamma-induced protein 10 (IP-10), platelet-derived growth factor (PDGF)-BB, macrophage inflammatory protein (MIP)-1α, and VEGF. 

### 3.6. Statistical Analysis 

Independent *t*-tests (equal variances not assumed), one-way ANOVA *post-hoc* Bonferroni, or non-parametric data analysis using Spearman rank correlation and Wilcoxon tests were used to assess levels and changes in BOP and cytokine concentrations. 

## 4. Conclusions 

This short-term study shows a dose dependent beneficial effect of bilberry intake on local markers of inflammation. Daily intake of 500 g of bilberries resulted in a clinically relevant reduction of gingival inflammation as well as reduction in GCF levels of pro-inflammatory levels of cytokines. The inclusion of a placebo group identified that participation in a study of this nature can result in important gingival changes. It appears that berry intake reduce gingival inflammation to the same extent as “standard of care”, that is routine debridement and oral hygiene instructions.
